# Optical Coherence Tomography Angiography Parameters of the Retina in SARS-CoV-2 Recovered Subjects

**DOI:** 10.7759/cureus.33548

**Published:** 2023-01-09

**Authors:** Punita K Sodhi, Ritu Arora, Suresh Kumar, Kirti Jaisingh, Archana T R., Kavya C Rao, Karan Chhabra, Sonal Saxena, Vikas Manchanda, Shantanu Sharma

**Affiliations:** 1 Ophthalmology, Guru Nanak Eye Centre and Maulana Azad Medical College, New Delhi, IND; 2 Medicine, Maulana Azad Medical College, New Delhi, IND; 3 Ophthalmology, All India Institute of Medical Sciences, Jodhpur, IND; 4 Internal Medicine, Beaumont Hospital, Royal Oak, USA; 5 Microbiology, Maulana Azad Medical College, New Delhi, IND; 6 Community Medicine, Mamta Health Institute for Mother and Child, New Delhi, IND

**Keywords:** indian population, covid-19, sars-cov-2, choroid, retina, vessel density, foveal avascular zone, optical coherence tomography angiography (oct-a)

## Abstract

Introduction: This study aims to evaluate retinochoroidal optical coherence tomography angiography (OCTA) parameters in patients recovered from severe acute respiratory syndrome coronavirus 2 (SARS-CoV-2).

Methods: This study was an observational study that included 80 subjects being discharged after having negative reports on the reverse transcription-polymerase chain reaction (RT-PCR) test for SARS-CoV-2 to evaluate OCTA parameters of the retina. The subjects underwent an ophthalmic evaluation that included best-corrected visual acuity (BCVA), intraocular pressure (IOP), color vision (CV), contrast sensitivity (CS), and optical coherence tomography (OCT) parameters. OCTA was done for all patients and was evaluated for foveal avascular zone (FAZ) area, perimeter, and circularity index, and vessel density (VD) in superficial capillary plexus (SCP), deep capillary plexus (DCP), outer retina (OR), outer retina chorio-capillaries (ORCC), chorio-capillaries (CC), and choroid (C) using 3 x 3 mm scans. The OCTA parameters were compared with normative data of the Indian population for various parameters in question.

Results: The subjects included 54/80 (67.5%) males and 26/80 (32.5%) females having a mean age of 52.40 ± 15.71 (18-60) years. The systemic evaluation revealed 38.75% of subjects had hypertension, 30% had diabetes, 20% had kidney disease, 5% had tuberculosis, and 3.75% had coronary artery disease. The mean distance BCVA was logarithm of the minimum angle of resolution (LogMAR) (1.17 ± 0.22), mean IOP was 17.0 ± 4.0 mmHg, mean CS was 2.13 ± 0.36, 50.62% of subjects had normal CV on Farnsworth test while 47% had tritanopia, and none of the subjects had red-green CV defect on Ishihara plates. The OCT scan was normal in 90% of eyes while the posterior vitreous detachment was seen in 4% of eyes, broad vitreomacular adhesion in 2.5% of eyes, and the globally adherent epiretinal membrane was seen in 2.5% of eyes. The mean central macular thickness (CMT) measured 245.14 ± 28.41 micrometers. The mean FAZ area measured 0.37 ± 0.15 mm^2^, the perimeter was 3.28 ± 1.08 mm, and the circularity index measured 0.41 ± 0.10. The average VD in SCP measured 16.06 ± 12.29, in DCP measured 9.11 ± 8.75, in OR measured 6.38 ± 7.37, in ORCC measured 42.53 ± 12.46, in CC measured 25.83 ± 16.31, and in C measured 25.52 ± 17.49. The VD in coronavirus disease 2019 (COVID-19) subjects was significantly lesser than that in the healthy Indian population in all layers except ORCC.

Conclusions: The SARS-CoV-2 recovered subjects have a reduced VD in retinochoroidal layers from COVID-19, an underlying systemic disease, or both. The CS values fall within normal limits. Several subjects show tritanopia on the Farnsworth test but no red-green CV defect on Ishihara plates.

## Introduction

In the retina, angiotensin-converting enzyme 2 (ACE-2) receptors are present in endothelial cells, Muller’s cells, ganglion cells, photoreceptors, and retinal pigment epithelium. These receptors may act as the potential sites of severe acute respiratory syndrome coronavirus 2 (SARS-CoV-2) affection in the retina [1]. The vascular endothelium has a vital role in controlling vascular tone and preserving the blood-retinal barrier [2], and its injury may lead to vasoconstriction and microvascular ischemia [2,3]. The immune cell and complement debris increase the risk for thromboembolism [4,5]. Moreover, there is a significant elevation of vascular endothelial growth factor-D (VEGF-D), cytokines, and inflammatory factors specifically seen in patients with ocular symptoms like conjunctivitis, critically ill patients, and those admitted to intensive care unit (ICU) [6-8].

An in vivo, non-invasive, microvascular function assessment is possible only in a few organs, including skin, bulbar conjunctiva, sublingual mucosa, and retina [3,9]. In the retina, optical coherence tomography angiography (OCTA) can clearly delineate the foveal avascular zone (FAZ) and quantify the blood flow in different capillary plexi, including superficial capillary plexus (SCP) and deep capillary plexus (DCP), which form the blood-retinal barrier, and other choroidal layers as well [3,10].

Though retinal macrovasculature (central retinal artery and vein) occlusion has been well described in the literature in patients with coronavirus disease 2019 (COVID-19) [11-14], only a few studies have described microvasculature affection of the retina [3,15-17]. OCTA is an invaluable tool to detect retinochoroidal microvascular abnormalities even before the onset of clinically evident retinopathy [10]. The OCTA parameters on FAZ and vessel density (VD) in COVID-19 subjects have been rarely reported [3,15-17]. Previous studies have shown that OCTA parameters may be used as surrogate biomarkers for varied microvascular changes occurring in different body systems [3]. These authors have recommended further studies to confirm their findings and thus highlight OCTA’s role in managing COVID-19 patients for ocular and other organ systems affection [3].

We evaluated OCTA parameters of the retina in SARS-CoV-2 recovered subjects.

This study was originally presented as a physical poster at the 80th All India Ophthalmological Society Conference 2022, Mumbai, India, on June 04, 2022.

## Materials and methods

We conducted this observational study for one year at the Department of Ophthalmology following approval by the Institutional Ethics Committee, Maulana Azad Medical College vide letter number F1/IEC/MAMC/(80/08/2020/No.256) (dated: October 16, 2020). The subjects in the age range of 18-60 years who had undergone treatment/monitoring for SARS-CoV-2 disease and were discharged after having negative reports on reverse transcription-polymerase chain reaction (RT-PCR) test for SARS-CoV-2 in nasopharyngeal swabs were enrolled following informed consent. The subjects were examined within two weeks of their discharge. The subjects having COVID-19 of mild to moderate severity were included. Exclusion criteria included significant ocular pathologies like retinal diseases, uveitis, glaucoma, high refractive error, central nervous system diseases, and ocular and cranial surgery other than uneventful cataract surgery or treatment with retinotoxic drugs. Systemic diseases like diabetes, hypertension, and coronary artery disease were recorded.

A thorough history was taken for symptoms like a diminution of vision, redness, irritation, foreign body sensation, tearing, and chemosis. The visual acuity was measured from ETDRS (Early Treatment Diabetic Retinopathy Study) charts in LogMAR (log of minimal angle of resolution) units followed by refraction. Keeping the contagious and recurrent nature of infection and propensity to spread through ocular secretions, we measured the intraocular pressure (IOP) with non-contact tonometry (Shin-Nippon, NCT-10, Indo Meditech, New Delhi, India). We looked for signs (cells, flare, and keratic precipitates) of anterior segment inflammation and uveitis on the slit lamp. A dilated fundus examination was done with both direct and indirect ophthalmoscopy for cup-to-disc ratio and abnormalities like exudates, hemorrhages, capillary non-perfusion (CNP), and vessel abnormalities. A color fundus photograph was taken on a fundus camera (FF450plus Fundus Camera Visucam PRO NM, Carl Zeiss Meditec AG, Jena, Germany). The patients were studied for color vision (CV) on Ishihara color plates (38th edition, 2012) and the Farnsworth D-15 test. They were examined for contrast sensitivity (CS) on Pelli-Robson charts (Haag-Streit, Mason, OH). A spectral domain optical coherence tomography (SD-OCT) scan (Model RS 3000 Advance, Nidek, Fremont, CA) of central 30 degrees around the fovea was done to look for retinal and choroidal changes and central macular thickness (CMT). The CMT was measured at the fovea as the distance between the retinal pigment epithelium and the internal limiting membrane.

A 3 x 3 mm OCTA macular scan (Model RS 3000 Advance, Nidek, Fremont, CA) centered on the fovea was used to delineate the FAZ and to assess VD in different retinochoroidal layers including SCP, DCP, outer retina (OR), outer retina chorio-capillaries (ORCC), chorio-capillaries (CC), and choroid (C). The equipment performed 85,000 A-scans/second and used a light source of 880 nm. The OCTA analysis divided the macular region into fovea, parafovea, perifovea, and the whole image in each vascular network of the retina, according to the ETDRS classification of diabetic retinopathy [18]. The VD was defined as the percentage area occupied by the large vessels and microvasculature in these particular regions. The OCTA scans were done for all patients to look for features like enlarged FAZ, CNP, hemorrhage, and neovascularization (NV) at the macula. All images were taken at a signal strength index of 90%, scans were taken three times, and readings were averaged.

The fundus fluorescein angiography (FFA) was done by injecting 3 ml of 20% fluorescein dye intravenously, to look for leaks, CNP, and staining, wherever indicated.

The primary outcome parameters were OCTA parameters and the secondary outcome parameters were optical coherence tomography (OCT) parameters.

We compared the parameters of COVID-19 patients with those of healthy Indian subjects from the published literature [19].

Sample size calculation

According to the available literature (Wu et al.) [8], the incidence of ocular involvement is 31.6%. To have a confidence level of 95% and a power of study of 80%, we got a sample size of 84. We examined 160 eyes of 80 subjects in this study.

Statistical analysis

Statistical analysis was performed using SPSS version 25.0 software (IBM Corp., Armonk, NY). Qualitative data were expressed in frequency and percentage. For quantitative data, mean ± standard deviation was calculated for analysis. The normality of the data distribution was tested using the Kolmogorov-Smirnov test. For non-parametric data, median values and interquartile range (IQR) were also calculated. Depending on normality distribution, the difference between the mean of the two groups was compared by Student's t-test (for parametric data) or Mann-Whitney U test (for non-parametric data). A value of p < 0.05 was considered statistically significant.

## Results

The present study is an observational study conducted in the Department of Ophthalmology in 160 eyes of 80 consecutive subjects, meeting our inclusion criteria, for evaluating OCTA features in SARS-CoV-2 recovered subjects. The subjects had been admitted to the hospital with symptoms like fever, cough, breathlessness, headache, weakness, sore throat, and chest pain and were diagnosed to have mild to moderate severity of COVID-19. Supportive therapy with oxygen was given in 65% of subjects. None of them required admission to the ICU.

Among 80 subjects, there were 54/80 (67.5%) males and 26/80 (32.5%) females. The mean age of our subjects was 52.40 ± 15.71 (18-60) years. None of the subjects complained of diminution of vision, redness, irritation, foreign body sensation, tearing, or chemosis. A positive history of underlying disease, namely, hypertension was present in 31/80 (38.75%) subjects, diabetes in 24/80 (30%) subjects, kidney disease in 16/80 (20%) subjects, chronic liver disease in 6/80 (7.5%) subjects, chronic obstructive pulmonary disease in 6/80 (7.5%) subjects, hypothyroidism in 6/80 (7.5%) subjects, tuberculosis in 4/80 (5%) subjects (one of them having Pott’s spine), coronary artery disease in 3/80 (3.75%) subjects, and anemia in 2/80 (2.5%) subjects. Additionally, 2/80 (2.5%) patients had lymphoma, 1/80 (1.25%) had cerebrovascular disease (stroke), 1/80 (1.25%) had a seizure disorder, 1/80 (1.25%) had associated sepsis, 1/80 (1.25%) had asthma, while 24/80 (30%) subjects did not have any systemic disease. The mean distance best-corrected visual acuity (BCVA) was the logarithm of the minimum angle of resolution (LogMAR) (1.17 ± 0.22). The near BCVA ranged from N5-N36 (mean value was 8.97 ± 5.66). The spherical equivalent refractive error for distance ranged from -2.5 dioptres to 2.25 dioptres while the mean value was 0.35 ± 1.28 dioptres. The fundus examination showed a cup-to-disc ratio (C:D) ranging from 0.3:1 to 0.5:1 (0.40 ± 0.08), and a C:D ratio equal to more than 0.5:1 was seen in 66.25% of our subjects. The mean IOP in our subjects was 17.0 ± 4.0 mmHg (range: 10-21 mmHg). The anterior segment examination showed a normal iris and normal anterior chamber and no cells, flare, or abnormal contents.

In the 1-21 plates category, the mean number of Ishihara plates read correctly was 20.74 ± 0.86 (15-21 plates). The number of eyes able to read all 21 plates was 142 (88.8%), the number of eyes able to read 20 plates was four (2.5%), the number of eyes able to read 19 plates was 11 (6.9%), the number of eyes able to read 18 plates was one (0.6%), and the number of eyes able to read 15 plates was two (1.3%). In the 22-25 plates category, the mean number of plates read correctly was 4 ± 0 as all the eyes read all four plates; thus none of the subjects had red-green CV defect (CVD). On the Farnsworth test, 81 eyes (50.62%) had a normal CV, 28 eyes (17.5%) had mild tritanopia, 26 eyes (16.25%) had moderate tritanopia, 21 eyes (13.12%) had severe tritanopia, two eyes (1.25%) had deuteranopia, one eye (0.62%) had protanopia, and one eye (0.62%) had an irregular CVD defect. The mean CS measurement taken from Pelli-Robson charts was 2.13 ± 0.36 (0.25 to 2.3).

The SD-OCT scan was normal in 144 eyes (90%) while a posterior vitreous detachment was seen in six eyes (4%), broad vitreomacular adhesion in four eyes (2.5%), the globally adherent epiretinal membrane was seen in four eyes (2.5%), vitreous opacities in two eyes (1.25%), drusen in one eye (0.6%), blunt foveal contour in one eye (0.6%), hard exudates in one eye (0.6%), macular edema in one eye (0.6%), and fibrinoid pigment epithelial detachment in one eye (0.6%). None of the eyes had neurosensory detachment. The mean CMT measured 245.14 ± 28.41 (196-418) micrometers.

The OCTA scan showed an enlarged FAZ in 16 eyes and both distorted and enlarged FAZ in four eyes. The SCP showed reduced vascularity in 45 eyes with uniform distribution, in two eyes supero-temporal to the fovea, in two eyes both supero-temporal and infero-temporal to the fovea, in two eyes in the nasal SCP, and in three eyes in parafoveal SCP. In comparison, there was increased vascularity in nine eyes. There was a uniformly reduced vascularity in the DCP in 48 eyes and in parafoveal DCP in four eyes. There was a reduced VD in the choroid in two eyes and a reduced VD in all retinochoroidal layers in two eyes.

**Figure 1:**
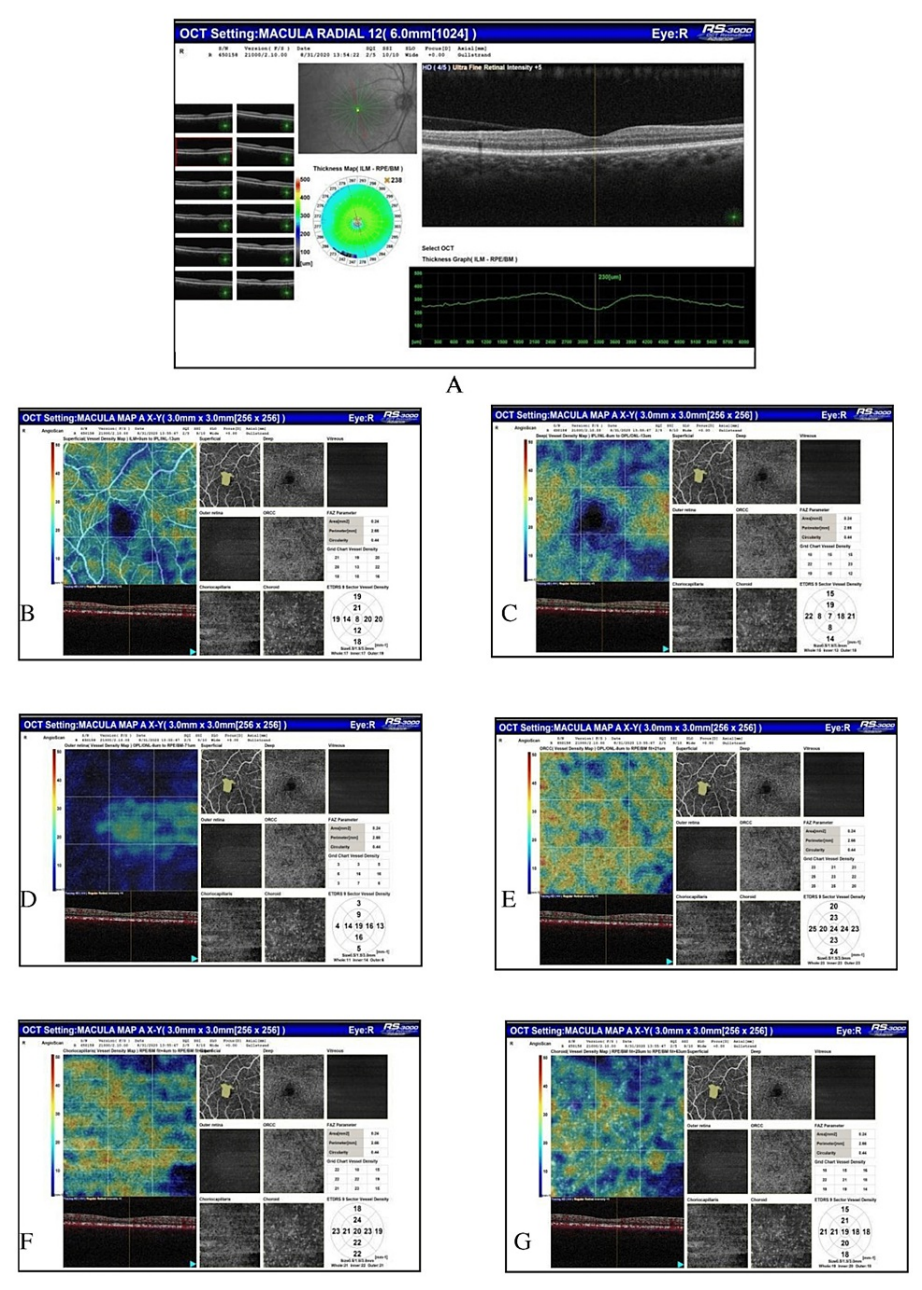
Optical coherence tomography of the right eye showing broad vitreomacular adhesion and central foveal thickness = 230 micrometers. Optical coherence tomography angiography showing foveal avascular zone (FAZ) area = 0.24 mm2, FAZ perimeter = 2.14 mm, FAZ circularity = 0.47, and vessel density of retinochoroidal layers including superficial capillary plexus, deep capillary plexus, outer retina, outer retina chorio-capillaries, chorio-capillaries, and choroid

Figure 1 shows the OCT and OCTA scans of a patient with broad vitreomacular adhesion and a central foveal thickness of 230 micrometers.

The FFA was indicated in one diabetic subject having dilated retinal vessels but only microaneurysms were seen on FFA.

We studied FAZ and VD in different retinochoroidal layers including SCP, DCP, OR, ORCC, CC, and C on quantitative OCTA scans. The normal values of these OCTA parameters were taken for comparison from published literature for healthy Indians [19]. The mean age of COVID-19 subjects in the present study was 52.40 ± 15.71 years while that of healthy Indian subjects in the published literature was 32.92 ± 9.14 years [19].

Table 1 presents the difference between mean values of ocular, OCT, and OCTA parameters of normal and COVID-19 subjects.

**Table 1:** Difference between mean values of ocular, OCT, and OCTA parameters of normal and COVID-19 subjects * Non-parametric data. COVID-19: coronavirus disease 2019; SD: standard deviation; CMT: central macular thickness; OCT: optical coherence tomography; OCTA: optical coherence tomography angiography; FAZ: foveal avascular zone; VD: vessel density; SCP: superficial capillary plexus; DCP: deep capillary plexus; OR: outer retina; ORCC: outer retina chorio-capillaries; CC: chorio-capillaries.

S. No.	Parameters	Mean ± SD (range) in healthy subjects	Mean ± SD (range) in COVID-19 subjects	Mean difference	P-value (t-test)
1	Intraocular pressure (mmHg)	17.0 ± 1.8 (14 to 20)	17.21 ± 4.55 (10 to 21)	0.21	0.7
2	Distance refractive error (in spherical equivalent dioptres)	0.08 ± 0.74 (-1 to +1)*	0.35 ± 1.28 (-2.5 to+2.25)*	0.27	<0.01
3	CMT (microns)	237.5 ± 26.0 (184 to 340)	245.14 ± 28.41 (196 to 418)	7.58	<0.01
4	FAZ area (mm^2^)	0.42 ± 0.23 (0.02 to 2.04)*	0.37 ± 0.15 (0.02 to 0.82)*	0.05	0.12
5	FAZ perimeter (mm)	3.3 ± 1.0 (0.68 to 7.71)	3.28 ± 1.08 (0.49 to 7.9)	0.02	0.43
6	FAZ circularity index	0.46 ± 0.1 (0.21 to 0.71)	0.41 ± 0.10 (0.17 to 0.74)	0.04	<0.01
7	VD SCP foveal	5.20 ± 7.19 (0 to 52)*	4.08 ± 6.53 (0 to 56)*	1.12	0.13
8	VD SCP parafoveal	26.61 ± 13.42 (1 to 50.5)*	14.40 ± 0.58 (0 to 44)	12.21	<0.001
9	VD SCP perifoveal	39.81 ± 15.83 (6 to 56)*	23.52 ± 16.02 (1.5 to 56)*	16.28	<0.001
10	VD SCP whole	23.87 ± 10.66 (2.33 to 47.3)*	16.06 ± 12.29 (0.6 to 45)*	7.81	<0.001
11	VD DCP foveal	2.71 ± 5.68 (0 to 48)*	1.76 ± 4.02 (0 to 33)*	0.95	0.01
12	VD DCP parafoveal	15.79 ± 11.34 (0 to 49)*	7.67 ± 7.23 (0 to 31)*	8.11	<0.001
13	VD DCP perifoveal	29.59 ± 16.45 (0 to 55.25)*	14.65 ± 13.04 (0 to 54)*	14.93	<0.001
14	VD DCP whole	16.03 ± 9.90 (0 to 40.33)*	9.11 ± 8.75 (0 to 38)*	6.91	<0.001
15	VD OR foveal	13.58 ± 14.79 (0 to 59)*	7.01 ± 10.22 (0 to 63)*	6.57	<0.001
16	VD OR parafoveal	16.89 ± 16.05 (0 to 64.75)*	8.27 ± 9.90 (0 to 53)*	8.61	<0.001
17	VD OR perifoveal	9.20 ± 8.71 (0 to 34.75)*	4.68 ± 6.04 (0 to 34)*	4.51	<0.001
18	VD OR whole	13.22 ± 12.27 (0 to 48.58)*	6.38 ± 7.37 (0 to 43)*	6.83	<0.001
19	VD ORCC foveal	37.75 ± 17.54 (0 to 69)*	37.95 ± 16.11 (6 to 69)*	0.2	0.86
20	VD ORCC parafoveal	39.71 ± 14.41 (0 to 62.75)*	40.61 ± 12.83 (10 to 62)	0.9	0.7
21	VD ORCC perifoveal	41.76 ± 14.04 (9 to 59)*	45.70 ± 13.23 (12 to 56)	3.93	<0.001
22	VD ORCC whole	39.74 ± 14.32 (6 to 60.33)*	42.53 ± 12.46 (11 to 57)	2.79	0.35
23	VD CC foveal	37.15 ± 18.42 (0 to 69)*	26.13 ± 18.11 (0 to 66)*	11.01	<0.001
24	VD CC parafoveal	37.02 ± 16.85 (0 to 65)*	25.85 ± 16.42 (1.25 to 57)*	11.16	<0.001
25	VD CC perifoveal	36.88 ± 16.12 (3 to 57)*	26.11 ± 15.98 (1 to 55)*	10.76	<0.001
26	VD CC whole	37.02 ± 16.43 (2.67 to 61.6)*	25.83 ± 16.31 (2.4 to 56)*	11.17	<0.001
27	VD choroid foveal	38.10 ± 18.48 (0 to 66)*	26.91 ± 18.86 (0 to 68)*	11.18	<0.001
28	VD choroid parafoveal	36.92 ± 16.62 (0 to 60)*	25.16 ± 17.61 (3.25 to 56)*	11.76	<0.001
29	VD choroid perifoveal	37.27 ± 16.92 (2 to 56.25)*	25.15 ± 17.63 (3.25 to 54)*	12.1	<0.001
30	VD choroid whole	37.43 ± 16.76 (1 to 59.33)*	25.52 ± 17.49 (2.8 to 55)*	11.91	<0.001

Table 1 shows that there was a statistically significant difference between COVID-19 and normal subjects with respect to mean values of distance refractive error, CMT, FAZ circularity index, VD SCP parafoveal, VD SCP perifoveal, VD SCP whole, VD DCP foveal, VD DCP parafoveal, VD DCP perifoveal, VD DCP whole, VD OR foveal, VD OR parafoveal, VD OR perifoveal, VD OR whole, VD ORCC perifoveal, VD CC foveal, VD CC parafoveal, VD CC perifoveal, VD CC whole, VD choroid foveal, VD choroid parafoveal, VD choroid perifoveal, and VD choroid whole.

As large amounts of data were non-parametric, the median and IQR were also calculated and the difference was found between values. Table 2 presents the difference between median values of ocular, OCT, and OCTA parameters of normal and COVID-19 subjects.

**Table 2:** Difference between median values of ocular, OCT, and OCTA parameters of normal and COVID-19 subjects COVID-19: coronavirus disease 2019; IQR: interquartile range; CMT: central macular thickness; OCT: optical coherence tomography; OCTA: optical coherence tomography angiography; FAZ: foveal avascular zone; VD: vessel density; SCP: superficial capillary plexus; DCP: deep capillary plexus; OR: outer retina; ORCC: outer retina chorio-capillaries; CC: chorio-capillaries.

S. No.	Parameters	Median and IQR in healthy subjects	Median and IQR in COVID-19 subjects	P-value (Mann-Whitney test)
1.	Intraocular pressure (mmHg)	17.0 (16.0 to 18.0)	17.3 (14.6 to 19.7)	0.05
2.	Distance refractive error (in spherical equivalent dioptres)	0.50 (-0.5 to 0.75)	0.25 (0.0 to 1.0)	0.69
3.	CMT (microns)	235 (217 to 257)	242 (225.7 to 262)	0.01
4.	FAZ area (mm^2^)	0.38 (0.30 to 0.47)	0.36 (0.27 to 0.46)	0.53
5.	FAZ perimeter (mm)	3.1 (2.6 to 3.6)	3.2 (2.6 to 3.9)	0.91
6.	FAZ circularity index	0.47 (0.39 to 0.53)	0.39 (0.35 to 0.48)	<0.001
7.	VD SCP foveal	3.0 (1.0 to 7.0)	2.0 (0.0 to 5.0)	0.48
8.	VD SCP parafoveal	29.7 (14.0 to 38.2)	13.0 (7.9 to 16.7)	<0.001
9.	VD SCP perifoveal	48.5 (21.0 to 53.7)	18.7 (13.9 to 28.0)	<0.001
10.	VD SCP whole	27.3 (12.4 to 32.5)	11.6 (8.1 to 19.0)	<0.001
11.	VD DCP foveal	0.0 (0.0 to 3.0)	0.0 (0.0 to 1.0)	0.01
12.	VD DCP parafoveal	13.0 (7.2 to 22.2)	7.0 (1.2 to 12.0)	<0.001
13.	VD DCP perifoveal	27.0 (16.2 to 46.2)	11.5 (4.7 to 20.8)	<0.001
14.	VD DCP whole	13.9 (9.0 to 23.8)	7.2 (2.1 to 12.0)	<0.001
15.	VD OR foveal	8.0 (2.0 to 19.0)	3.0 (1.0 to 9.0)	<0.001
16.	VD OR parafoveal	11.5 (4.8 to 25.7)	5.5 (2.0 to 11.0)	<0.001
17.	VD OR perifoveal	6.0 (2.7 to 13.0)	2.2 (1.0 to 7.0)	<0.001
18.	VD OR whole	8.6 (3.3 to 20.6)	3.5 (1.6 to 9.0)	<0.001
19.	VD ORCC foveal	40 (22.2 to 53.7)	39.0 (24.0 to 52.0)	0.34
20.	VD ORCC parafoveal	46 (22.8 to 50.6)	45 (35 to 49.7)	0.364
21.	VD ORCC perifoveal	50 (24 to 53.2)	53 (41 to 54)	0.001
22.	VD ORCC whole	45.6 (23.2 to 52.0)	47.5 (41.0 to 51.0)	0.015
23.	VD CC foveal	40 (20.0 to 54.0)	19 (14 to 44.7)	<0.001
24.	VD CC parafoveal	41.2 (20.2 to 52.0)	19.1 (15.7 to 46.0)	<0.001
25.	VD CC perifoveal	44.1 (20.0 to 51.5)	19.5 (16.4 to 43.7)	<0.001
26.	VD CC whole	43.0 (20.1 to 51.9)	18.3 (15.2 to 46.7)	<0.001
27.	VD choroid foveal	45.0 (17 to 54)	16.5 (13.2 to 48.0)	<0.001
28.	VD choroid parafoveal	45.8 (17 to 50.2)	15.5 (12.5 to 47.0)	<0.001
29.	VD choroid perifoveal	47.8 (16.8 to 51.2)	15.2 (12.7 to 47.7)	<0.001
30.	VD choroid whole	45.1 (17.1 to 51.6)	15.2 (13.0 to 49.0)	<0.001

There was a statistically significant difference between COVID-19 and normal subjects for all features except IOP, distance refractive error, FAZ area, FAZ perimeter, VD SCP foveal, VD ORCC foveal, and VD ORCC parafoveal. The values in COVID-19 subjects were higher for IOP, CMT, FAZ perimeter, VD ORCC perifoveal, and VD ORCC whole while lesser for the rest of the features.

## Discussion

A wide range of clinical manifestations of COVID-19, including pneumonia, cerebrovascular disease, and multiorgan failure, has made it vital to examine the underlying pathophysiology [3]. As the retina is an extension of the brain, an evaluation of retinochoroidal vasculature can provide information on multi-organ ischemic sequelae including the brain to find the impact of the COVID-19 virus [3]. The retina is one of the most perfused organs and is resistant to the influence of autonomic innervation and hormones. Even during changes in blood pressure or blood gas tension, it can maintain a constant blood flow. These features make the retina an ideal organ to study the local microcirculation and its changes [20]. OCTA is an invaluable tool to detect microvascular abnormalities even before the onset of clinically evident retinopathy. Nevertheless, data on OCTA parameters in COVID-19 subjects has been rarely reported.

The prevalence of ocular manifestations in patients with COVID-19 ranges from 2% to 32%. These include conjunctivitis, uveitis, retinovascular, and neuro-ophthalmic disease [4]. Retinal ischemia has been observed in the form of central retinal artery and vein occlusion, retinal hemorrhages, cotton wools spots, dilated veins, and tortuous vessels, and notably, these can occur even in the absence of systemic vascular risk factors [11,12,14]. Acute macular neuro-retinopathy (AMN) and paracentral acute middle maculopathy (PAMM) result from deep retinal capillary plexus ischemia [13]. Invernizzi and colleagues measured retinal vein diameter at 0.5 to 1 DD from the optic disc margin using an automated retinal image analyzer and found that retinal vein diameter correlated directly with disease severity, suggesting that this may be a non-invasive parameter to monitor inflammatory response and/or endothelial injury in COVID-19 [14].

Several authors have studied the retina of COVID-19-affected subjects. Table 3 shows the findings of retinal examination with multimodal imaging including FFA, OCT, and OCTA of COVID-19 patients in different studies.

**Table 3:** Findings of retinal examination, FFA, OCT, and OCTA in COVID-19 patients * No significant difference from control subjects. FFA: fundus fluorescein angiography; OCT: optical coherence tomography; OCTA: optical coherence tomography angiography; COVID-19: coronavirus disease 2019; CWS: cotton wool spots; SCP: superficial capillary plexus; DCP: deep capillary plexus; OR: outer retina; ORCC: outer retina chorio-capillaries; CC: chorio-capillaries; ERM: epiretinal membrane; MH: macular hole; CMT: central macular thickness.

Authors	Fundus findings	FFA	OCT/equipment	OCTA
Markan et al. [5]	CWS (5.38%), intra-retinal hemorrhages (5.38%)	Not mentioned	At sites corresponding to CWS “hyper-reflective sign” and cyst in the acute stage, and inner retinal atrophy in later stages	De-correlation signal (reduced vascularity) in all layers including SCP, DCP, OR, and CC; flow signal got restored on the resolution of CWS
Marinho et al. [21]	CWS (33%), micro-hemorrhages along the retinal arcade (33%)	Not mentioned	Hyper-reflective lesions at the level of ganglion cells and inner plexiform layers	Normal parameters but specific names of parameters not mentioned
Invernizzi et al. [14]	Hemorrhages (9.25%), CWS (7.4%), dilated veins (27.7%), tortuous vessels (12.9%)	Not mentioned	Dilated retinal arteries and veins on an automated retinal image analyzer	Not mentioned
Savastano et al. [16]	CWS (12.9%), vitreous degeneration (14.28%), hemorrhage (1.42%)	Not mentioned	ERM (7.1%), MH (7.1%), CMT 263.7 ± 25.68 microns*	Reduced VD and enlarged FAZ
Gonzalez-Zamora et al. [3]	CWS (20%)	Not mentioned	CMT 241.1 ± 3.77 microns*	Reduced VD and enlarged FAZ
Turker et al. [17]	Not mentioned	Not mentioned	Not mentioned	Reduced VD and enlarged FAZ; increased VD in CC
Abrishami et al. [15]	No abnormality seen	Not mentioned	No abnormality seen	Reduced VD and enlarged FAZ
Our study	Dilated retinal vessels (1.25%)	Microaneurysms	CMT 245.14 ± 28.41 micrometers; OCT findings are given in the results section	Reduced VD except for ORCC and reduced FAZ circularity

The COVID-19 patients in our study had a higher incidence of systemic diseases like those of Savastano et al. [16]. These authors did not find a significant difference (p = 0.34) in mean IOP between control and affected subjects [16]; we made a similar observation. Like other authors [15,16,21], we did not observe signs of intraocular inflammation.

We examined subjects of COVID-19 virus infection for OCTA parameters including FAZ and VD in different retinochoroidal layers to detect vascular flow abnormalities.

Table 4 shows the difference in demography and OCTA parameters between COVID-19 and healthy subjects observed in different studies.

**Table 4:** Difference in demography and OCTA parameters between COVID-19 and healthy subjects observed in different studies OCTA: optical coherence tomography angiography; COVID-19: coronavirus disease 2019; M: males; F: females; FAZ: foveal avascular zone; VD: vessel density; SCP: superficial capillary plexus; DCP: deep capillary plexus; CC: chorio-capillaries; C: COVID-19 subjects; N: healthy controls.

	Authors	COVID-19 patients	Healthy subjects	Difference	P-value
Number of subjects	Turker et al. [17]	27 (14M and 13F)	27 (14M and 13F)		
	González-Zamora et al. [3]	25 (14M and 11F)	25 (14M and 11F)		
	Abrishami et al. [15]	31 (17M and 14F)	23 (14M and 9F)		
	Savastano et al. [16]	70 (39M and 31F)	22 (8M and 14F)		
	Our study	80 (54M and 26F)			
Mean age (years)	Turker et al. [17]	38.74 ± 10.70	37.44 ± 10.04		0.648
	González-Zamora et al. [3]	61.02 ± 1.64	59.62 ± 1.61		0.69
	Abrishami et al. [15]	40.4 ± 9.2	36.6 ± 7.1		0.115
	Savastano et al. [16]	53.7 ± 14	44.7 ± 11.3		0.006
	Our study	52.40 ± 15.71	32.92 ± 9.14		0.001
Type of subjects	Turker et al. [17]	Hospitalized patients			
	González-Zamora et al. [3]	Bilateral pneumonia			
	Abrishami et al. [15]	Mild			
	Savastano et al. [16]	Mild to moderate			
	Our study	Mild to moderate			
Time of examination after discharge	Turker et al. [17]	7 days			
	González-Zamora et al. [3]	14 days			
	Abrishami et al. [15]	14 days			
	Savastano et al. [16]	30 days			
	Our study	14 days			
OCTA parameters					
SCP VD	Turker et al. [17]	49.85 ± 4.88 superior	51.82 ± 3.37 superior	C less than healthy subjects	0.017
	Turker et al. [17]	47.75 ± 4.29 nasal	49.99 ± 4.01 nasal	C less than healthy subjects	0.006
	González-Zamora et al. [3]	15.2 ± 0.69	29.1 ± 1.88	C less than healthy subjects	<0.001
	Abrishami et al. [15]	44.98 ± 4.16	48.36 ± 2.24	C less than healthy subjects	0.001
	Savastano et al. [16]	21.20 ± 1.2	21.20 ± 1.4	C less than healthy subjects	0.99
	Our study	16.06 ± 12.29	23.87 ± 10.66	C less than healthy subjects​​​​​​​	<0.001
DCP VD	Turker et al. [17]	39.49	54.86	C less than healthy subjects​​​​​​​	0.01
	González-Zamora et al. [3]	13.33 ± 0.93	19.77 ± 1.53	C less than healthy subjects​​​​​​​	0.001
	Abrishami et al. [15]	49.74 ± 3.39	53.03 ± 3.29	C less than healthy subjects​​​​​​​	0.001
	Savastano et al. [16]	21.82 ± 2.51	23.05 ± 3.44	C less than healthy subjects​​​​​​​	0.88
	Our study	9.11 ± 8.75	16.03 ± 9.90	C less than healthy subjects​​​​​​​	<0.001
FAZ area (mm^2^)	Turker et al. [17]	0.30 ± 0.14	0.29 ± 0.11	C > N	0.493
	González-Zamora et al. [3]	0.29 ± 0.25	0.19 ± 0.26	C > N	0.007
	Abrishami et al. [15]	0.27 ± 0.11	0.24 ± 0.08	C > N	0.191
	Savastano et al. [16]	0.24 ± 0.11	0.24 ± 0.09	C > N	0.98
	Our study	0.37 ± 0.15	0.42 ± 0.23	C less than healthy subjects​​​​​​​	0.12
FAZ perimeter	Savastano et al. [16]	2.04 ± 0.52	2.06 ± 0.36	C less than healthy subjects​​​​​​​	0.87
	Our study	3.28 ± 1.08	3.3 ± 1.0	C less than healthy subjects​​​​​​​	0.43
CC VD	Turker et al. [17]	2.15 ± 0.23	2.08 ± 0.11	C > N	0.042
	González-Zamora et al. [3]	49.1 ± 1.01	50.3 ± 1.03	C less than healthy subjects​​​​​​​	0.40
	Our study	25.83 ± 16.31	37.02 ± 16.43	C less than healthy subjects​​​​​​​	<0.001

We found FAZ area and perimeter to be lesser in COVID-19 subjects dissimilar to that reported earlier [3,15-17]. Savastano et al. also found a lesser value for FAZ perimeter in COVID-19 subjects [16]. The oxygen therapy causes hyperoxia and vasoconstriction; these may cause an enlargement of FAZ and a reduction in VD of SCP and DCP [17]. González-Zamora et al. felt that these may be due to thrombotic and inflammatory phenomena in the retina of COVID-19 subjects [3].

The COVID-19 subjects had lesser VD in SCP and DCP than healthy subjects [3,15-17]. Rather, we found the VD of COVID-recovered subjects to be significantly lesser than that of the healthy Indian population in all layers except ORCC. The lesser affection of VD of ORCC in COVID-19 subjects was probably due to the supply of this layer from the CC plexus, which is a highly anastomosed capillary network. Even though González-Zamora et al. saw a lower value for VD in the CC layer of COVID-19 subjects compared to healthy controls, they felt that the CC layer is not significantly compromised due to disease as it has a highly anastomosed and dense capillary network [3]. Turker et al. made an opposite observation. The ischemia of choroid tissue leads to local hypercapnia, vasodilation, and an increased CC flow [17]. Additionally, systemic factors of inflammation also increase choroidal blood flow as the choroid is not autoregulated like the retina [9].

This difference in the affection of FAZ and VD may be due to the fact that factors influencing VD may be slightly different from those affecting the distribution of vessels, i.e., the distance at which vessels stop at the margin around the fovea. Our patients had COVID-19 of mild to moderate severity and the FAZ area and perimeters may be parameters more amenable to recovery after the resolution of the acute phase of infection. An absence of macular microvascular impairment in mild to moderately affected subjects was observed by Savastano et al. also and they suggested that this damage may be found in severely affected patients [16].

Our study had a larger sample as compared to previous studies. We also studied features like refractive error, intraocular pressure, color vision, and contrast sensitivity. The limitation of our study was that there was non-availability of OCTA data at the time of acute infection and admission of patients. Thus, the role of COVID-19 vis-a-vis underlying diseases in affecting OCTA features cannot be elucidated. Additionally, the severely affected ICU patients could not attend the ophthalmology department within two weeks of their discharge. The FF450plus fundus camera provided only a central 55-degree fundus view and our equipment provided the OCTA scans measuring 3 x 3 mm. Thus, the periphery of the retina could not be examined.

In patients hospitalized in the ICU for COVID-19, the incidence of venous thromboembolic phenomena is about 25%. The authors have seen this incidence despite anticoagulant prophylaxis. The retinal circulation is an end arterial system, thus retinal vascular diseases are potentially vision-threatening [11]. As the retina is an extension of the brain, thus retinochoroidal vascular findings in the form of an enlarged FAZ and a reduced VD could be associated with similar changes in the brain resulting in CNS manifestations and neurological events in COVID-19 [3,20]. These OCTA parameters may indicate the severity of COVID-19 disease and help to tailor personalized therapies for patients to improve prognosis.

## Conclusions

We found that many of our mild to moderately affected COVID subjects fell into a higher age group and had an underlying systemic disease. The distance BCVA affection might be due to an older age, underlying diseases, COVID-19, or a cumulative effect of all three factors. The CS did not appear to be reduced and the subjects did not have red-green CV defects when tested on Ishihara plates. About half of the subjects showed tritanopia on the Farnsworth test. The FFA and OCT scans were predominantly normal. The FAZ area and perimeter were not very severely affected at two weeks following discharge, though the FAZ circularity index was significantly reduced. A significantly reduced VD in retinochoroidal layers might be due to COVID-19 or the underlying systemic disease or both.
